# Established and emerging treatments for eating disorders

**DOI:** 10.1016/j.molmed.2024.02.009

**Published:** 2024-04

**Authors:** Callum Bryson, Daire Douglas, Ulrike Schmidt

**Affiliations:** 1 Institute of Psychiatry, Psychology, and Neuroscience, King’s College London, London, UK; 2 South London and Maudsley NHS Foundation Trust, London, UK

**Keywords:** eating disorders, treatment, neuromodulation, virtual reality, psychedelics, metreleptin

## Abstract

Eating disorders (EDs) are common mental health conditions that carry exceedingly high morbidity and mortality rates. Evidence-based treatment options include a range of psychotherapies and some, mainly adjunctive, pharmacological interventions. However, around 20–30% of people fail to respond to the best available treatments and develop a persistent treatment-refractory illness. Novel treatments for these disorders are emerging, but their efficacy and clinical relevance need further investigation. In this review article, we first outline the evidence-base for the established treatments of the three ‘classical’ EDs [anorexia nervosa (AN), bulimia nervosa (BN), and binge eating disorder (BED)]. We then review research on some of the most promising emerging treatment modalities, discussing the questions and challenges that remain.

## Highlights

Current eating disorder treatments include a range of psychological interventions, such as cognitive behavioural therapy, family therapy, or more structured specialised treatments such as Maudsley model of anorexia treatment for adults (MANTRA), and some off-label pharmacological interventions.
Even with best available treatments, remission rates remain suboptimal and many patients go on to develop long-term treatment-refractory disorders. It is, therefore, crucial that novel treatments are developed, in attempts to significantly speed up and improve remission rates.
Neuromodulation, virtual reality therapy, psychedelics, and leptin analogues are among emerging treatments that may help improve patient outcomes.

## Eating disorders: treatment and challenges

Feeding and EDs are severe psychiatric disorders with complex biopsychosocial origins [1]. Among these, AN, BN, and BED are most well-known (Box 1). Other EDs include Other specified feeding or eating disorders (OSFED), pica, rumination disorder, and avoidant-restrictive food intake disorder (ARFID), which are all characterised by pathological eating behaviours. EDs represent some of the highest mortality and morbidity rates among mental health conditions, underscoring the gravity of these conditions [2].

Box 1 **Overview of anorexia nervosa, bulimia nervosa, and binge eating disorder**

AN is characterised by an intense fear of weight gain and body image disturbances, leading to extreme food intake restriction, weight loss, and low body weight. Individuals with AN may also engage in compensatory behaviours such as excessive exercise, self-induced vomiting, or laxative misuse. AN can result in a range of physical symptoms, including hormonal disturbances, amenorrhea, gastrointestinal problems, or hair loss.

BN is recognised by two main features: episodes of uncontrolled overeating (bingeing) followed by harmful compensatory behaviours to control weight gain (purging). Individuals with BN are often normal weight or overweight. Purging behaviours often result in physical symptoms such as dental erosion, salivary gland swelling, bloating, and indigestion, in addition to those associated with food restriction, such as in AN.

BED is characterised by frequent episodes of uncontrolled consumption of large amounts of food (bingeing) in short periods of time, usually followed by feelings of guilt, shame, and embarrassment. Unlike BN, BED does not involve compensatory behaviours. BED usually occurs in individuals who are normal weight, overweight, and obese. Physical symptoms are generally linked to effects of overeating, notably weight gain, low energy or digestion, and bowel problems, as well as a lack of nutrient consumption depending on the patient’s overall diet.

These disorders are also frequently associated with comorbid mental health conditions such as depressive, bipolar and anxiety disorders, obsessive compulsive disorder (OCD), or substance abuse [1].

Alt-text: Box 1

Treatment guidelines emphasise outpatient psychological therapies as primary interventions for EDs [3]. For adolescents, **family-focused treatments** (see Glossary) are recommended, while adults may undertake **cognitive behavioural therapy (CBT)**, guided self-help approaches, **Maudsley model of anorexia treatment for adults (MANTRA)**, **specialist supportive clinical management (SSCM)**, or **focal psychodynamic therapy (FPT)**. Treatments may also involve nutritional interventions and multimodal day- and inpatient treatments in more severe cases. **Pharmacological therapies** are often used in adjunct to psychological interventions, particularly for BN and BED. Antidepressants, antipsychotics, and stimulants, such as lisdexamfetamine dimesylate (henceforth lisdexamfetamine), are among pharmacotherapeutic options.

**Figure 1:**
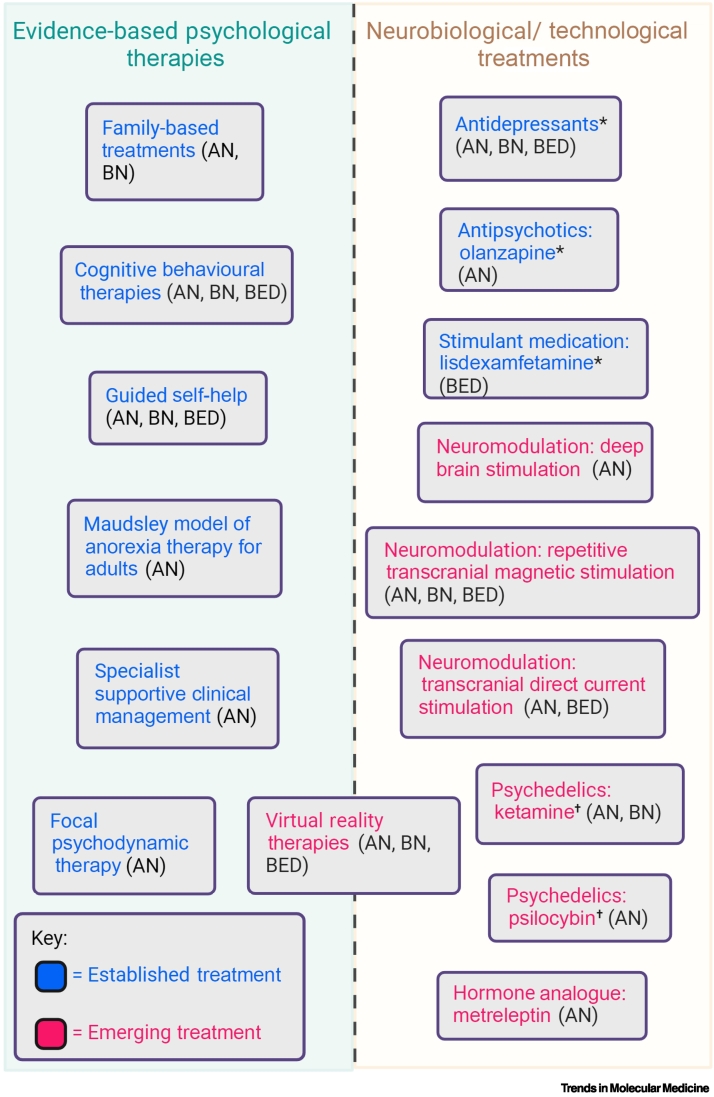
An illustration of the types of eating disorder (ED) treatments and their novelty. The schematic illustrates the different types of ED treatments that are discussed in the review. The left-hand column refers to established evidence-based psychological therapies, while the right relates to neurobiological and/or technological treatments. Virtual reality therapies are placed in between the two columns to acknowledge that these treatments apply common principles from psychological therapies in a novel technology. The letters in brackets refer to the ED diagnoses that these interventions are recommended/used for, in the case of the established treatments, or are the topic of current research for the emerging treatments. Finally, ‘*’ is used to denote the established treatments that are recommended to be used as adjuncts to psychological therapies, while ‘†’ is used to denote the emerging treatments that have been designed with the intention to assist psychological therapies. This figure was created using (https://biorender.com/). Abbreviations: AN, anorexia nervosa; BED, binge eating disorder; BN, bulimia nervosa.

Even with these best available evidence-based interventions, ED recovery rates are still suboptimal, with estimates that 20–30% of patients with AN or BN do not respond to treatments and develop long-term **treatment-refractory illnesses**, motivating the development of a range of novel interventions [2,4]. This review aims to provide a synthesis of established psychological and pharmacological treatments and emerging treatments for AN, BN, and BED (see Clinician’s corner). ‘Established treatments’ refers to evidence-based interventions recommended in clinical guidelines that are widely adopted in practice, while emerging treatments that are explored in this article include **neuromodulation**, virtual reality (VR) therapy, psychedelics, and metreleptin (Figure 1). While this list is by no means comprehensive, these treatments represent what we consider to be the key established and most promising emerging treatments in the field at this time. These four emerging treatment modalities were chosen in part due to their larger evidence base compared with other new treatments and in part to demonstrate a range of novel approaches. Thus, our choice has a degree of subjectivity. Finally, due to a lack of recent literature summarising the current landscape of ED treatments, this review seeks to offer an updated overview of treatment options and help to direct efforts to the most promising emerging treatments while identifying key issues in their delivery.

## Established treatments for eating disorders

### Psychological therapies

Guidelines agree that the majority of patients with EDs should be treated with structured psychological therapies [5]. Treatment approaches are usually divided into those for children and adolescents and those for adults, due to different developmental needs. For adolescents with AN, family-focused treatments seem to be advantageous over individual therapies, be that conjoint family therapy or multifamily groups [6]. Brief formats of family-focused treatments seem to work well except for those from single-parent families or where the patient has obsessive compulsive disorder (OCD) symptoms [7]. Separate family therapy for parents and patients seems to work better for families with high expressed emotion [8], and adding intensive parental coaching to standard family-focused treatments may improve outcomes where parents have low self-efficacy [9]. Guided self-help formats have also been trialled with some success in the USA [10], but not in Australia [11], highlighting the importance of implementation context. Finally, family approaches are recommended for adolescents with BN, with CBT approaches as an alternative.

Treatments for adults with AN usually involve individual outpatient therapies, such as CBTs for EDs (CBT-ED), MANTRA, SSCM, or FPT [12]. Outcomes across these different treatments seem to be similar, potentially due to overlaps in strategies used for behaviour change and the application of more intensive measures for those who do not respond [13].

The recommended first-line treatment for adults with BN or BED is an ED-specific form of CBT, which is superior to alternative psychological interventions [14]. Guided self-help formats of CBT, delivered via books or online, or briefer forms (CBT-10) are also effective starter treatments in stepped care services and can be as effective as face-to-face CBT [15].

In recent years, there has been a blurring of boundaries between treatments for adolescents and for adults, in recognition of the fact that emerging adults (age 18–25 years) are often closely involved with and reliant on their families for support. Thus, family approaches are increasingly trialled in emerging adult populations too, with positive effects [6]. Conversely, individual ED therapies such as CBT-ED or MANTRA are increasingly being adapted to younger adolescent populations by inclusion of some sessions with parents [16,17]. Finally, a recent rapid review suggested that there is growing evidence supporting the use of **third wave psychological therapies** as first-line treatments for EDs, however, additional studies are needed to determine their efficacy [18].

While the psychological therapies discussed earlier are considered to be the most effective treatments for EDs, they have several key limitations that contribute to low recovery rates [2]. Namely, several factors are known to influence the effectiveness of these treatments, with poorer patient outcomes being observed in those with a longer illness duration, lower motivation for change, greater presence of comorbidities, more severe ED psychopathology, and poorer interpersonal functioning and relationships [19,20]. Additionally, help-seeking and recognition of EDs is variable across diagnoses, which limits access to these therapies, especially amongst men and minoritised ethnic groups [21].

### Pharmacological treatments

Antidepressants were introduced as off-label treatments for EDs due to the high levels of comorbid depressive, anxious, and obsessive-compulsive symptoms [22]. Where antidepressant use is recommended, they should be used as adjuncts to psychological therapy and not as a sole treatment. This is likely due to the multifaceted nature of these disorders, whereby pharmacological interventions cannot address some key illness mediators (i.e., psychosocial factors), the lower acceptability of medications compared with psychological therapies, and the potential impact of side effects [14,23].

In AN, treatment guidelines largely advise against the use of antidepressants [2,22]. Tricyclic antidepressants and monoamine oxidase inhibitors are strongly discouraged due to their limited efficacy and safety concerns, including increased seizure and cardiovascular risk and lack of safety when taken in overdose [3,23]. Despite a more favourable safety profile, selective serotonin reuptake inhibitors (SSRIs), such as fluoxetine or sertraline, have also failed to elicit improvements in weight or AN symptomatology; however, there is some limited evidence that their use (adjunctive to psychotherapies) may assist with relapse prevention [3,23., 24., 25., 26.]. Mirtazapine, a tetracyclic antidepressant, has a weak recommendation based on two studies demonstrating improvements in weight and mood [3,27,28]; however, further research into its efficacy is warranted. Health professionals are advised to exercise clinical judgement when considering antidepressants for patients with AN, as despite inconclusive results, the combination of treatments may act synergistically to promote and maintain recovery in some patients, particularly those with severe comorbid disorders [23].

For adults with BN and BED, the SSRI fluoxetine is approved and recommended due to its effectiveness in reducing bingeing and purging behaviours, with early evidence for its efficacy and safety in younger patients [3,29., 30., 31., 32.]. While, overall, antidepressants are regarded as safe and acceptable for use in BN and BED treatments, the limited efficacy data available for both disorders leads to only weak recommendations in guidelines [3,29]. This is, with the exception of bupropion, an aminoketone antidepressant that significantly elevates seizure risk in those with purging behaviours, making it contraindicated in patients with EDs [33,34].

Antipsychotics are routinely prescribed off-label for AN, with the most widely used being olanzapine, because of its appetite enhancing effect. A systematic review and meta-analysis of six trials found that in adolescents and adults with AN, olanzapine had a small effect on weight gain when given as an adjunct to treatment, but its effect on psychopathology is less clear [35]. Of course, many patients with AN do not want to take such a medication due to weight gain fears.

Lisdexamfetamine, originally used as a stimulant medication for attention-deficit/hyperactivity disorder, is approved in North America for patients with BED [29,36., 37., 38.]. Doses of 30, 50, and 70 mg have been shown to more greatly reduce bingeing frequency and general ED psychopathology compared with both placebo and active controls [14,39,40]. The medication is available to patients who either prefer drug-based interventions, or who cannot access or do not respond to psychotherapy [29]. While lisdexamfetamine is known to have potential serious cardiovascular adverse events [41], it has been shown to have comparable safety and tolerability in BED and ADHD populations [42]. Further research should be directed towards investigating these safety concerns, focusing on the suitability for long-term use in BED populations.

Overall, current evidence shows some SSRIs, lisdexamfetamine, and olanzapine to be effective and, largely, safe in reducing some ED psychopathology and/or comorbid symptoms, although longer term effects are more uncertain.

## Emerging treatments for eating disorders

As noted, recovery rates for those with EDs remain low with best available treatments. This may be due to the complex interplay of factors underlying these illnesses, with growing evidence of neurobiological, metabolic, endocrine, and genetic contributions, alongside the better recognised psychosocial factors [43]. Current psychological and pharmacological treatments may only address a limited number of contributing factors [44], perhaps the reason for low recovery rates. Consequently, there is recognition in this field of the need for novel therapeutic options for patients with EDs, many of which are attempting to target alternative contributing factors of AN, BN, and BED. This section will explore the use of neuromodulation, VR therapy, psychedelics, and metreleptin to treat AN, BN, and BED, focusing on literature from the past ~5 years or earlier where evidence is limited.

### Neuromodulation

Improved understanding of the neurocircuitry involved in EDs has given rise to the use of electromagnetic neuromodulation treatments, including **deep brain stimulation (DBS)**, **repetitive transcranial magnetic stimulation (rTMS)**, and **transcranial direct current stimulation (tDCS)**. Most available evidence focuses on severe, enduring, and/or treatment-refractory AN, although noninvasive forms of neuromodulation (tDCS, rTMS) are increasingly used in people with BN or BED.

AN is the only ED where the use of DBS has been explored, usually in patients with very long-lasting and treatment-refractory illness. A systematic review of 17 studies included a total of 118 adolescent and adult patients with AN who underwent stimulation to the sub-callosal cingulate (SCC) in 50% of cases and the nucleus accumbens (NAcc) in around 30% of cases [45]. Overall, patients exhibited body mass index (BMI) improvements of around 25% over, approximately, a 17-month follow-up period. Adverse effects included post-operative and post-stimulative seizures (9% prevalence), electrolyte disturbances (5%), and infection (1%). A further meta-analysis of four studies with a total of 56 adult treatment-refractory patients analysed outcomes after DBS to the NAcc (32 patients), SCC (20 patients), and ventral anterior limb of the interior capsule (vALIC; four patients) [46]. There were improvements in BMI, mood, anxiety, OCD symptoms, ED symptoms, and quality of life at follow-up (6–24 months). Adverse events occurred in nine cases (e.g., hypomania or seizure). Finally, a systematic review and network meta-analysis for 36 cases of DBS in AN identified the SCC as the stimulation site most strongly associated with BMI change, compared with the NAcc and vALIC [47].

A **sham-controlled**, **feasibility** randomised controlled trial (RCT) examined the use of **neuro-navigated**, high frequency rTMS to the dorsolateral prefrontal cortex (DLPFC) in adult outpatients with treatment-refractory AN, reporting early improvements in mood, life quality, and food choices, with later improvements in BMI [48,49]. Importantly, changes in amygdala regional blood flow correlated with later BMI improvements. In BN, two small RCTs of rTMS had negative findings, showing no superiority of rTMS over sham [50,51]. Interestingly, in an ancillary study to [50], improvements in inhibitory control and decision-making, but not in clinical measures, were observed post-treatment [50,52]. This may suggest that rTMS improves neurocognitive functioning, which then leads to clinical improvements, but the threshold for clinical improvement was not reached in this study. Future research could investigate the relationship between these factors to determine if, for example, improvements in neurocognitive functioning precede clinical improvements during rTMS. Finally, in BED, one double blind RCT of 60 adults receiving 20 sessions of rTMS to the left DLPFC reported significant improvements in bingeing episodes, but not in food cravings or mood, in both the real and sham groups (M.F. Maranhão *et al*., unpublished data).

A double-blind sham-controlled RCT examined the use of ten sessions of left DLPFC tDCS in adult inpatients with AN [53]. No significant effects on ED psychopathology or weight recovery were observed; however, as participants in both conditions were receiving inpatient refeeding, this may have obscured any tDCS effects. However, tDCS did seem to reduce patients’ need to follow specific dietary rules and improved body image evaluation. Three sham-controlled trials in adults with BED augmented tDCS with different types of cognitive training, showing encouraging results [54,55] (G. Gordon, PhD thesis, King’s College London, 2021). The largest of these trials had four parallel groups (total *n* = 82): attention bias modification training (ABMT) alone, ABMT plus real or sham tDCS, and waiting list [55]. At the end of treatment and follow-up, there was a clear advantage of combination treatment in terms of greater reduction in binges, ED symptoms and weight, and improvements in mood. Clinical trials into the use of tDCS for patients with BN are lacking.

The evidence demonstrates strong potential for the safe and tolerable use of neuromodulation in AN and BED. Further effort should be directed towards understanding the potential of these techniques in patients with BN. Finally, key questions remain about the use of neuromodulation as a stand-alone versus adjunctive therapy, as well as the exact neural mechanisms underpinning the observed therapeutic effects.

### VR therapy

VR is a computer-based technology that can generate interactive and immersive environments that simulate the real world [56]. These environments can be paired with traditional therapeutic strategies, such as cue exposure therapy or CBT, to enhance their efficacy [57]. By providing controllable and modifiable environments for therapy, VR offers patients with EDs the opportunity to interface with troubling scenarios that would be difficult to reproduce in the real world [58]. As a result, this may enable patients to more readily alter their body cognitions or eating behaviours [59]. For example, patients can be exposed to virtual kitchens or restaurants where they can simulate seeing, touching, and/or, eating a variety of foods. Alternatively, participants may be shown a virtual avatar of themselves, which slowly changes size and shape to match that of their target weight over several sessions. For a more detailed review of types of VR therapies, see [60].

Only one small RCT has evaluated the efficacy of VR-based therapy in 35 adolescent and adult patients with AN [61]. Patients were provided with either five sessions of VR therapy adjunctive to usual treatment, or with usual treatment alone. In these sessions, participants were presented with a virtual avatar of their body, which, over the sessions, increased incrementally in BMI when patients showed a diminished anxiety response. The VR therapy was shown to be more effective at reducing body dissatisfaction and fear of weight gain at post-treatment, relative to the treatment as usual arm. These differences remained stable at follow-up.

In patients with BN, there is evidence to suggest that VR therapies can be effective. For example, in one RCT [62], 24 women with BN were offered either ten sessions of VR-enhanced CBT or a multimodal inpatient care programme. The VR sessions exposed participants to triggering stimuli in several virtual environments with the aim of practicing skills learnt in their CBT sessions. Patients provided with the VR therapy showed a decrease in their ED cognitions and behavioural symptoms at post-treatment and a lower frequency of binge and purge episodes at 9-months follow-up, relative to the control group. These findings have been corroborated by two other RCTs in adolescent and adult patients with EDs (98 patients across the studies, 52 of which had BN), which similarly found that VR-enhanced CBT was superior to regular CBT at improving patients’ body image and frequency of binge/purge episodes, alongside other ED behaviours, at post-treatment [63,64]. These differences were either stable or increased in magnitude at follow-up [65].

Three papers, with varying female BED samples and two to four treatment arms, all seem to emanate from one registered trial (ISRCTN59019572)i. It is not clear whether they refer to distinct or overlapping patient populations, but they present somewhat inconsistent results pertaining to the efficacy of VR treatments in adult patients with BED. In the first of these, 20 women with BED were treated with either a CBT method aimed at changing their maladaptive behaviours (psycho-nutritional group) or VR-enhanced CBT [66]. VR therapy was superior to psycho-nutrition at improving body satisfaction, self-efficacy, and motivation for change. The second of these papers demonstrated VR-enhanced CBT to be superior to CBT, psycho-nutrition, and waitlist conditions at improving body satisfaction and self-efficacy. VR was also superior at improving bulimia symptoms at 6-months follow-up, but not weight loss [67]. In the final paper, VR-enhanced CBT was more effective at maintaining weight loss at 1-year follow-up, relative to the three other conditions, but not for any other factor measured [68]. The reasoning behind these inconsistencies is unclear, especially as these three papers are from the same trial and share their methodology.

The literature suggests that VR therapies may be advantageous treatment for EDs over CBT. However, the VR stimuli, number of sessions, methods of evaluating attenuation to stimuli, and the use of adjunctive treatments varied significantly between studies. Moreover, most of these studies did not report the patients’ illness duration and/or comorbidities, or actively excluded participants with multiple diagnoses. Before VR therapies are implemented in clinical settings, replication studies are needed, alongside a standardisation and extension of their methods.

### Psychedelic drugs

Psychedelics are a class of psychoactive substances that induce profound alterations in perception and mood. A large body of literature has demonstrated psychedelics, notably ketamine and psilocybin, to have antidepressant effects in individuals with major depression, prompting investigation into their potential use in ED populations [69].

#### Ketamine

Ketamine is an *N*-methyl-d-aspartate (NMDA) receptor antagonist, traditionally used in anaesthesiology, and at sub-anaesthetic doses it induces dissociative effects. Research in depressed populations has established ketamine as a fast-acting but transient antidepressant [70].

As this treatment in EDs is in its infancy, only a few case studies and a singular open-label trial have explored its efficacy and tolerability among patients with AN and BN. The patients included in these studies were adults with long-lasting EDs, with the exception of one study involving adolescents, and all exhibited various comorbidities, including depression and anxiety disorders, OCD, trauma, or personality disorders [71., 72., 73., 74., 75., 76., 77., 78., 79.]. The ketamine interventions also varied in terms of dosage, administration methods, and the presence or type of additional treatments. Overall, studies reported improvements in mood and anxiety, with weak evidence of weight improvements and ED symptomatology reductions. Ketamine interventions were well-tolerated, with mild adverse effects typically occurring during treatment sessions and resolving quickly afterwards, consistent with findings in depressed populations [80]. RCTs are required to robustly evaluate the treatment potential of ketamine across ED diagnoses. In addition, existing studies investigate varying ketamine interventions, ranging from intramuscular or intranasal administration, esketamine (a ketamine derivative), and combinations with different therapies and diets. Controlled trials will assist in establishing more consistent protocols, including standardised dosage, treatment duration, and frequency.

#### Psilocybin

Psilocybin is a serotonergic compound, most notably found in the *Psilocybe* mushroom genus. While interest in psilocybin’s therapeutic potential in psychiatry is widespread, its use in ED populations is still limited [69].

An open-label feasibility study investigating psilocybin-assisted therapy in ten adult patients with AN reported good tolerability and safety, but variable efficacy, with four out of ten patients demonstrating reductions in ED symptomatology but no effect on BMI. [81]. The authors caution that due to the size and design of this study, results are preliminary.

### Metreleptin

Leptin is a hormone secreted by **adipose cells** that has a critical role in the homeostatic control of eating behaviours and weight [82]. Although this hormone is primarily involved in hypothalamic functions [83], it is also known to act on midbrain reward circuits and influence motivations towards food stimuli [84]. Importantly, aberrant leptin levels may be a key pathophysiological marker for EDs, from which symptoms later emerge [85,86]. As such, it has been suggested that leptin **analogues**, such as metreleptin, might be a useful treatment for these disorders, especially AN (for an in-depth review of the rationale, see [87]).

To date, four case studies have been published on the use of metreleptin to treat AN [88., 89., 90., 91.]. Of the six patients included, four were adolescents. The presence of comorbid diagnoses was not reported, with illness duration only being reported in two of the case studies and three of these four patients having an illness duration of more than 5 years. While metreleptin administration varied, most patients showed improvements in mood, ED symptom severity, and core ED cognitions during and immediately after their dosing period. Some patients were also observed as being more sociable and gained small amounts of weight over this period [88,91] and, where assessed, excessive exercise stabilised [88]. At follow-up, these improvements either remained stable or increased, with all patients having been stepped down to outpatient facilities or having a complete cessation of their symptoms.

While these findings are promising, no RCTs have been conducted to assess the efficacy of metreleptin to treat AN. This research is necessary to validate metreleptin as the cause of these improvements and to inform future clinical practice about, for example, the best treatment schedule for patients, the optimal patient subgroups for treatment, and if the treatment should be provided alone or adjunct to psychotherapy.

## Concluding remarks

Overall, there is some promising, albeit nascent, evidence to suggest that the novel interventions discussed in this review could be effective treatments for EDs. Notably, these interventions are in varying development stages, with neuromodulation treatments having a more substantive evidence base. Due to their novelty and recency, the emerging interventions require further investigation to establish efficacy in relation to other forms of available treatment and to identify optimal treatment protocols (see Outstanding questions). Moreover, more effort should be made to consider the efficacy of these treatments in patients with common comorbidities (such as mood or anxiety disorders, OCD, or neurodevelopmental disorders) or over the full spectrum of illness duration, especially in the VR and metreleptin literature where comorbidities and illness duration are routinely not reported. Efforts to understand the neural mechanisms underpinning these treatments would also enable improvements in their efficacy. In the literature, explanations surrounding the enhancement of neural plasticity tend to be favoured, however, this area requires substantially more research. Finally, ED researchers should consider how they conceptualise and measure ED recovery. Specifically, the field would benefit from a standardisation of their outcome measures to allow for greater ease when comparing the efficacy of these interventions.

It should be acknowledged that ED research is severely underfunded compared with that of other psychiatric disorders of similar prevalence and disease burden, with publications from this field being under-represented in high impact journals [92]. A larger and more definitive body of evidence for these emerging treatments is imperative to ensuring that this vulnerable patient population is better served both in research and clinically. We would like to emphasise that while the evidence base for these treatments is still growing, their potential for improving patient outcomes and quality of life should inspire hope in researchers, clinicians, and patients alike.

Clinician’s corner Effective evidence-based first line treatments for people with eating disorders (EDs) are available in the form of family-focused interventions for children and adolescents and a range of outpatient psychotherapies for adults, most prominently cognitive behavioural therapy (CBT). Guided self-help formats of CBT are useful first line interventions for bulimia nervosa (BN) and binge eating disorder (BED).

Even with the best available psychotherapies, a substantial number of patients do not make a full recovery and 20–30% develop a persistent and treatment-refractory form of illness.

There are a range of novel interventions, including most prominently noninvasive and deep brain stimulation (DBS), virtual reality (VR) treatments, and novel pharmacological interventions (psychedelics, metreleptin).

These treatments are at different stages of development and evaluation, ranging from being supported by randomised controlled feasibility and efficacy trials [repetitive transcranial magnetic stimulation (rTMS), transcranial direct current stimulation (tDCS), VR therapy] or by case studies and uncontrolled studies (ketamine, psilocybin, metreleptin).

Importantly, much more work needs to be done to: (i) understand mechanisms of action; (ii) determine efficacy, effectiveness, and cost-effectiveness of these novel interventions; and (iii) determine optimal intervention targets and treatment populations. These novel approaches, nonetheless, should give hope to those affected by persistent feeding and EDs that have not responded to conventional treatments.

Alt-text: Clinician’s corner

Outstanding questions How do emerging treatments, such as neuromodulation, VR, psychedelics, and metreleptin compare with established treatments for EDs in efficacy, effectiveness, and cost-effectiveness?

Should these treatments be delivered as stand-alone or adjunctive treatments?

What are the neural mechanisms underlying any improvements seen in EDs and other symptoms in any of these novel treatments?

Is neuromodulation targeting ED symptoms or comorbid depressive symptoms?

What are the optimal stimulation targets and protocols for neuromodulation treatments?

What are the optimal protocols for psychedelic and metreleptin therapeutic administration?

What modalities of VR therapies offer the most effective treatments for EDs?

Are these novel treatments equally effective across different ED diagnoses and illness stages?

Alt-text: Outstanding questions

## Glossary

**Adipose cells**: a type of connective tissue cell that stores energy in the form of fat.
**Analogue**: a synthetic substance that mimics the structure and function of another naturally occurring substance.
**Cognitive behavioural therapy (CBT)**: a goal-oriented therapy that addresses dysfunctional thought patterns and behaviours, promoting the development of healthy coping strategies.
**Deep brain stimulation (DBS)**: an invasive neuromodulatory technique that involves surgically implanting electrodes in specific brain regions to deliver controlled electrical impulses, modulating neuronal activity.
**Family-focused treatments**: different specialised treatments for adolescents with active family involvement, where parents are encouraged to engage with, take charge of, and support their child’s recovery either by working with the whole family, the parental couple, or multiple families.
**Feasibility**: a preliminary study, usually a clinical trial, conducted to assess the practicality, potential risks, and overall viability of a similar larger-scale project.
**Focal psychodynamic therapy (FPT)**: a therapeutic approach focused at gaining insight into underlying psychological and emotional issues contributing to the eating disorder, rooted in traditional psychodynamic principles.
**Maudsley model of anorexia treatment for adults (MANTRA)**: a cognitive-interpersonal treatment for adults with anorexia that considers the interplay of biology and psychology in the maintenance of the illness.
**Neuromodulation**: medical technology employing invasive and noninvasive procedures to modify brain activity electrically or chemically, utilised in disease treatment.
**Neuro-navigation**: computer-assisted technologies, such as structural magnetic resonance imaging (MRI) scans, used to identify and target specific brain regions during various treatments or surgical procedures.
**Pharmacological therapy**: the treatment of illnesses using medication.
**Repetitive transcranial magnetic stimulation (rTMS)**: a noninvasive neuromodulation technique that uses magnetic fields to induce electrical currents in targeted brain areas.
**Sham-controlled**: the use of a placebo or inactive intervention that mimics the real intervention (‘sham’), enabling researchers to evaluate the real efficacy of a treatment by comparing outcomes between active and ‘sham’ groups.
**Specialist supportive clinical management (SSCM)**: a treatment utilising supportive therapy techniques to improve patients’ quality of life and mood alongside addressing their eating disorder symptoms.
**Third wave psychological therapies**: contemporary approaches that incorporate methods and assumptions such as acceptance, mindfulness, psychological flexibility, or emotional regulation into traditional therapies.
**Transcranial direct current stimulation (tDCS)**: a noninvasive neuromodulation technique involving the application of low electrical currents to the scalp.
**Treatment-refractory illnesses**: illnesses that persist despite various treatment attempts.

## Resources

^i^ www.isrctn.com/ISRCTN59019572
